# Treatment of gouty arthritis is associated with restoring the gut microbiota and promoting the production of short-chain fatty acids

**DOI:** 10.1186/s13075-022-02742-9

**Published:** 2022-02-19

**Authors:** Han-Ki Park, Sang Jin Lee

**Affiliations:** 1 Department of Internal Medicine, School of Medicine, Kyungpook National University, Daegu, Republic of Korea; 2 Department of Molecular Medicine and Biopharmaceutical Sciences, Graduate School of Convergence Science and Technology, and College of Medicine, Medical Research Institute, Seoul National University, Seoul, Republic of Korea; 3 Department of Internal Medicine (Rheumatology), Kyungpook National University Hospital, 130 Dongdeok-ro, Jung-gu, Daegu, 41944 Republic of Korea

**Keywords:** Gouty arthritis, Gut microbiota, Short-chain fatty acids

## Abstract

**Introduction:**

Although factors initiating the inflammatory response to monosodium urate crystals have been identified, the role of the gut microbiota and their metabolites on gout remains unknown. This study aimed to investigate the changes in both gut microbiota and short-chain fatty acids (SCFAs) according to inflammatory states of gout in the same patients.

**Methods:**

This study enrolled 20 patients with gout in the acute state who had active joints and were followed up until the recovery state with no active joints. Blood and fecal samples were simultaneously collected within 3 days for each disease state. The stool microbiome was analyzed using 16S rRNA sequencing, and serum SCFAs were measured by gas chromatography-mass spectrometry. Differences in the gut microbiome and serum SCFAs were compared between the acute and recovery states.

**Results:**

Beta diversity of the microbiome was significantly different between the acute and recovery states in terms of weighted UniFrac distance. In the recovery state, Prevotellaceae (*p* = 0.006) and the genus *Prevotella* (*p* = 0.009) were significantly enriched, whereas Enterobacteriaceae (*p* = 0.019) and its derivative genus *Shigella* (*p* = 0.023) were significantly decreased compared to the acute state. Similarly, the levels of acetate were dramatically increased in the recovery state compared to the acute state (*p* < 0.010). The levels of propionate and butyrate tended to increase but without statistical significance.

**Conclusion:**

Substantial alterations of bacterial composition with the promotion of SCFA formation (especially acetate) were found after treatment in patients with gouty arthritis.

**Supplementary Information:**

The online version contains supplementary material available at 10.1186/s13075-022-02742-9.

## Introduction

Gout is a common disease of inflammatory arthritis which results from the inflammatory response to monosodium urate (MSU) crystals in the joints [1]. A secondary stimulus is required to develop acute gouty arthritis in individuals with hyperuricemia, which affects the deposition of MSU crystals in the joints [2]. Although factors modulating the acute inflammatory response to MSU crystals are better known, the effect of diet, gut microbiota, and metabolites on gout remains to be elucidated [3].

Short-chain fatty acids (SCFAs), which are produced by the gut microbiota that metabolize complex plant polysaccharides, have an important role in regulating immune cell function and the inflammatory response [4]. Acetate, one such SCFA, promotes the resolution of the inflammatory response to MSU crystals by inducing neutrophil apoptosis [5]. A drug with an anti-inflammatory response on gouty arthritis could possibly exert its therapeutic effect by affecting the gut microbiota and enhancing SCFA production in mice induced by MSU crystals [6]. These studies suggest that the gut microbiota and SCFAs are involved in modulating the inflammatory response to MSU crystal-induced arthritis; these warrant further research for the possible therapeutic effects in patients with gouty arthritis.

This study aimed to investigate the association of both gut microbiota and SCFAs with gouty arthritis. To understand the role of bacterial dysbiosis and SCFAs in its pathogenic mechanism, we analyzed the changes in the gut microbiota and SCFAs across the inflammatory states of gout in the same patients.

## Methods

### Patients and study design

This study enrolled 20 patients with active joints in the acute state who were followed up until the recovery state with no active joints at Kyungpook National University Hospital (KNUH) from August 2020 to May 2021. This study consisted of two different disease states, the acute state and recovery state, in the same patients. Blood and fecal samples were simultaneously collected within 3 days at each disease state in all patients. All samples were immediately stored at − 80 °C until analysis. Gout was diagnosed by a rheumatologist (S.J.L.) based on the American College of Rheumatology/European League Against Rheumatism criteria of 2015 [7], and enrolled patients were fitted to the definition of gout flare as having pain scores > 3 with a patient-reported gout flare [8]. The exclusion criteria were patients who were younger than 18 years old and those who were not followed up until the recovery state after the initial enrollment. No patients were offered any dietary modification that may have led to a change in the microbiota. The protocol was approved by the Institutional Review Board and the Ethics Committee at KNUH. The study was conducted in full accordance with the principles of the Declaration of Helsinki.

### Polymerase chain reaction (PCR) amplification of the bacterial 16S rRNA

DNA was extracted from feces using a DNeasy PowerSoil Kit (Qiagen, Hilden, Germany) according to the manufacturer’s instructions. The extracted DNA was quantified using Quant-IT PicoGreen (Invitrogen). The sequencing libraries are prepared according to the Illumina 16S Metagenomic Sequencing Library protocols to amplify the V3 and V4 regions. The input gDNA (2 ng) was PCR-amplified with 5× reaction buffer, 1 mM of dNTP mix, 500 nM each of the universal F/R PCR primer, and Herculase II fusion DNA polymerase (Agilent Technologies, Santa Clara, CA). The cycle condition for the 1st PCR was 3 min at 95 °C for heat activation and 25 cycles of 30 s at 95 °C, 30 s at 55 °C, and 30 s at 72 °C, followed by a 5-min final extension at 72 °C. The universal primer pair with Illumina adapter overhang sequences used for the first amplifications was as follows: V3-F 5′-GTCGGCAGCGTCAGATGTGTATAAGAGACAGCCTACGGGNGGCWGCAG-3′, V4-R 5′-GTCTCGTGGGCTCGGAGATGTGTATAAGAGACAGGACTACHVGGGTATCTAATCC-3′. The 1st PCR product was purified with AMPure beads (Agencourt Bioscience, Beverly, MA). Following purification, 2 μl of the first PCR product was PCR-amplified for the final library construction containing the index using the Nextera XT Indexed Primer. The cycle conditions for the second PCR were the same as that in the first PCR condition, except that it was only run for 10 cycles. The PCR product was purified with AMPure beads. The final purified product is then quantified using qPCR according to the qPCR Quantification Protocol Guide (KAPA Library Quantification kits for Illumina Sequencing platforms) and qualified using the TapeStation D1000 ScreenTape (Agilent Technologies, Waldbronn, Germany). The paired-end (2 × 300 bp) sequencing was performed by the Macrogen using the MiSeq™ platform (Illumina, San Diego, USA).

### SCFAs extraction and analysis using gas chromatography-mass spectrometry

To SCFAs, 100 μl of serum sample was mixed with 400 μl of 0.5% phosphoric acid solution and 500 μl of butanol. Then, 5.8 μl of 5800 ppm 4-methyl valeric acid was added as an internal standard. The mixture was homogenized and centrifuged (10 min, 13,000 rpm). The supernatant containing SCFAs was collected and stored until further analysis. Acetate, propionate, and butyrate (Sigma-Aldrich) were used for standard. The SCFAs were analyzed using gas chromatography-mass spectrometry (GC-MS) (Agilent 7820A, USA) and equipped with a DB-Wax column (50 m × 200 μm × 0.2 μm; Agilent Technologies). The GC oven condition was set to 90 °C, increased to 150 °C at 15 °C/min, increased to 250 °C at 5 °C/min, and then held for 0.25 min. Helium was used as a carrier gas at a constant flow rate of 1.5 ml/min. The GC-MS chromatograms were acquired using a scan mode of m/z 33–250 at a fragment voltage of 70 eV. Peaks were identified in the GC-MS chromatograms through a library search (NIST ver. 11) of their mass spectra.

### Processing of sequences and bioinformatics analysis

After sequencing of MiSeq raw data, a FASTQ file for each sample was created. The adapter sequence was removed using the Fastp program [9], and error correction was performed on the region where the two reads overlapped. The paired-end data for each sample was assembled into a single sequence using FLASH (v1.2.11) [10]. The resulting sequence was passed into CD-HIT-out [11], an operational taxonomic unit (OTU) analysis program based on CD-HIT-EST, to remove low-quality sequences, ambiguous sequences and chimera sequences, and clustering sequences with more than 97% sequence similarity to form a species-level OTU. The representative sequence of each OTU was performed by BLASTN (v.2.4.0) on the reference DB (NCBI 16S Microbial) [12], and the taxonomic assignment was performed with the organism information of the subject having the highest similarity. A variety of microbial community comparisons were performed using QIIME (v1.9) [13]. In order to check the species diversity and uniformity of the microbial community in the sample, alpha diversity information was confirmed through the rarefaction curve, Chao1 value, and Shannon index. Based on the weighted UniFrac distance, beta diversity between samples (information on diversity among samples in the comparison group) was obtained, and the relationship between samples was visualized through principal coordinate analysis (PCoA) and Heatmap. Linear discriminant effect size (LEfSe) analysis was performed to identify the bacteria that were significantly different; the degree of difference was expressed as a linear discriminant analysis (LDA) score with *α* = 0.05 and LDA score threshold-2. At this time, 0.5% or more of the genus level in at least 1 group was analyzed.

### Statistical analysis

Categorical variables are presented as their numerical value and percentage; these were analyzed using Pearson’s chi-squared test or Fisher’s exact test. Continuous variables are presented as mean ± standard deviation ranges and were analyzed using Student’s *t*-test or the Mann-Whitney *U*-test. Paired data were analyzed using the paired *t*-test. Pearson’s correlation coefficient was used to determine the correlations between continuous variables. All the results with *p* < 0.05 were considered statistically significant. Statistical analyses were performed using the SPSS software version 20.0 (IBM Corp., Armonk, NY, USA). The GraphPad Prism 9.0 software (GraphPad Inc., San Diego, CA, USA) was used to produce graphs.

## Results

### Characteristics of the enrolled gout patients

The fecal and serum samples were analyzed to assess the differences in the gut microbiota and SCFAs between acute and recovery states in the same patients with gouty arthritis (*n* = 20). Baseline characteristics of the enrolled patients are summarized according to different disease states in Table 1. The mean follow-up duration between acute and recovery states was 64.50 ± 25.66 days, and the majority of patients were male (95.0%). There were significant differences between both groups in terms of uric acid, erythrocyte sedimentation rates, and C-reactive protein levels. The first metatarsophalangeal (MTP) joint (8/20, 40.0%) was the most affected site, followed by the knee and ankle joint. All patients took colchicine, and 80% took urate-lowering agents during the acute state (Table 1).

**Table 1:** Characteristics of study participants with gouty arthritis according to disease states

Characteristics	Acute state	Recovery state
Age	63.90 ± 14.90^a^	
Sex, male (%)	19 (95.0)	
Follow-up duration, days	64.50 ± 25.66	
Uric acid	6.77 ± 1.96^†^	5.32 ± 1.74^†^
eGFR	71.90 ± 19.98	71.10 ± 16.83
ESR (mm/h)	49.94 ± 34.36^‡^	19.88 ± 19.50^‡^
CRP (mg/dl)	5.13 ± 6.90^§^	0.35 ± 0.54^§^
Involved joints at attack		
First MTP joint	8 (40.0)	
Knee/ankle	7 (35.0)/3 (15.0)	
Wrist/elbow	2 (10.0)/2(10.0)	
No. of involved joints at attack		
1	14 (70.0)	
2	1 (5.0)	
≥ 3	5 (25.0)	
Treatment agents, *n* (%)		
Colchicine	20 (100.0)	
NSAID	7 (35.0)	
Corticosteroid	4 (20.0)	
Intraarticular injection	9 (45.0)	
Urate lowering agents		
Febuxostat	14 (70.0)	
Allopurinol	1 (5.0)	
Benzbromarone	1 (5.0)	

Data are expressed as means ± SD for continuous variables or numbers and percentages for categorical variables

*eGFR* estimated glomerular filtration rates, *ESR* erythrocyte sedimentation rate, *CRP* C-reactive proteins, *MTP* metatarsophalangeal, *No.* number, *NSAID* non-steroidal anti-inflammatory drugs

^a^Of the 20 patients, two patients received antibiotics for 3 days at the time of acute state

^†^*n* = 19, *p* = 0.010

^‡^*n* = 17, *p* = 0.001

^§^*p* < 0.001

### Gut microbiota altered substantially in patients in the acute state

**Fig. 1:**
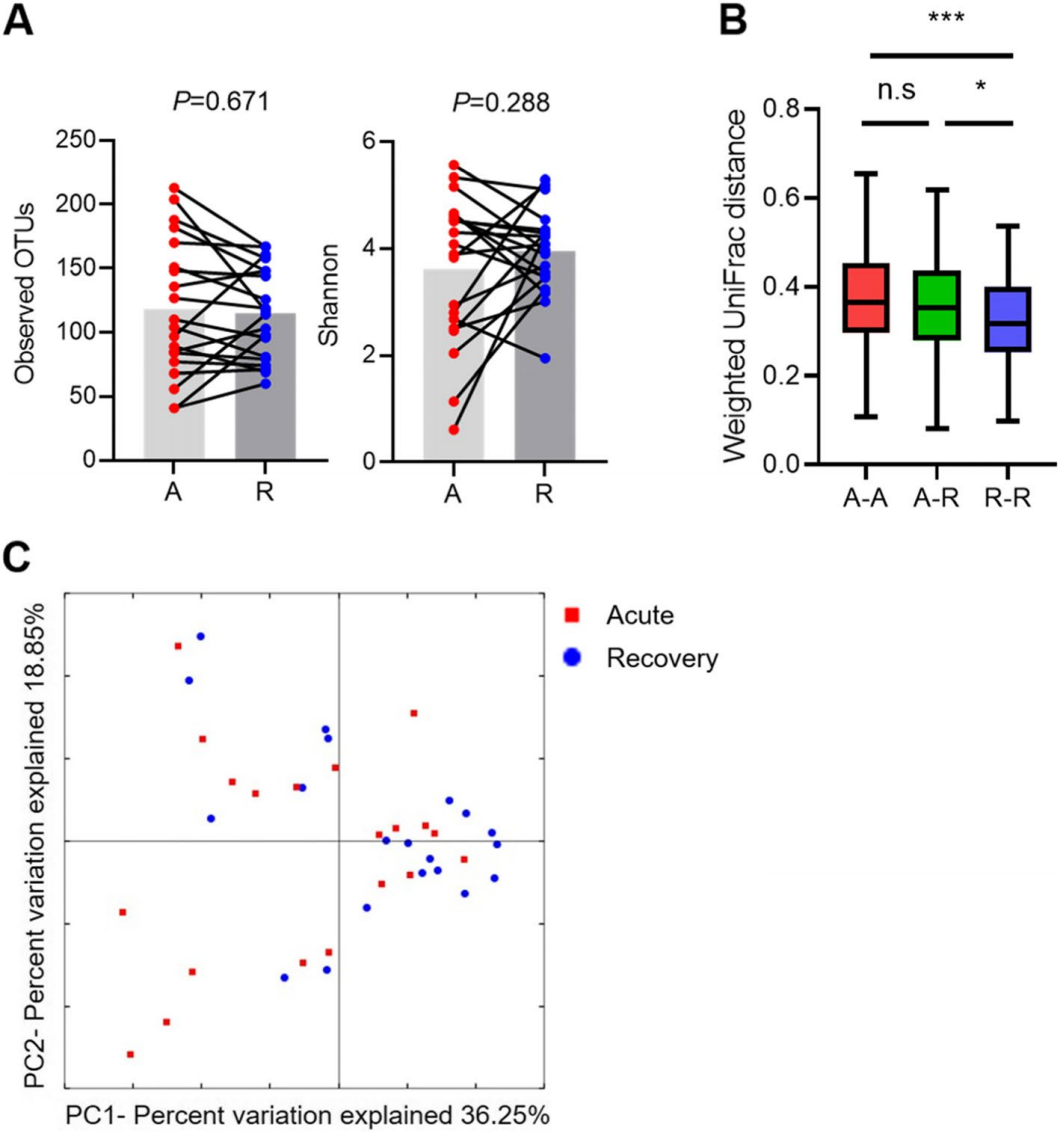
Gut microbiota composition between the acute state and recovery state. Alpha diversity (**A**). UniFrac distance based on weighted analysis (**B**). Principal coordinate analysis (PCoA) based on weighted analysis (**C**). A, acute state; R, recovery state; *P < 0.05; ***P < 0.001 (Student’s *t*-test)

**Fig. 2:**
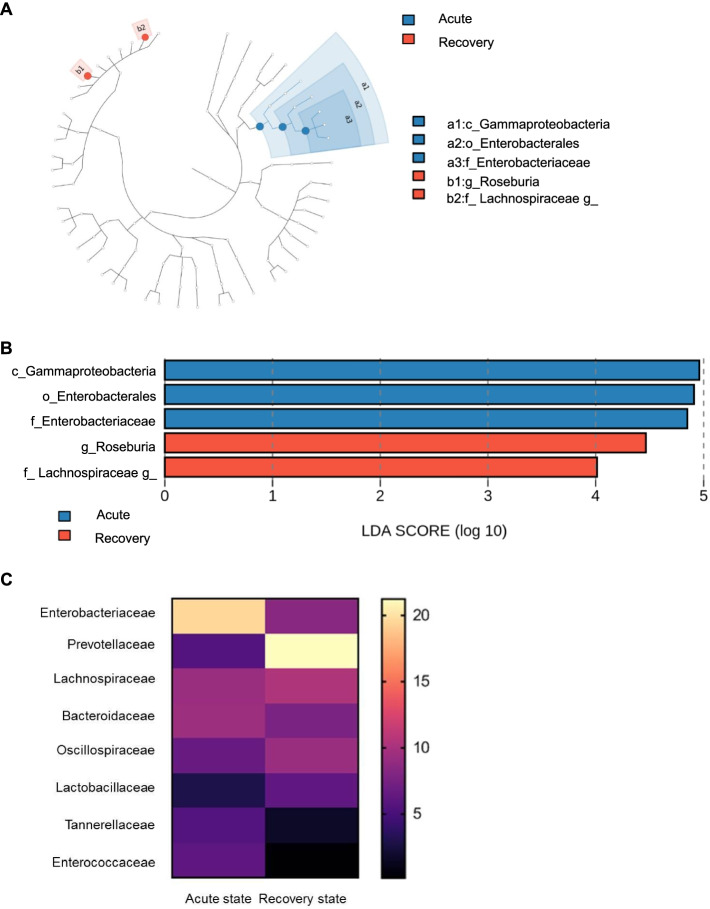
Relative abundances of the bacterial composition between acute state and recovery state of gout. Cladogram (**A**). Linear discriminant analysis (LDA) scores (**B**). Heatmap of differentially abundant families (**C**). The gut microbiota at the family level with more than 3% relative abundance were selected

The gut microbiota of recovery state patients in terms of alpha diversity (observed OTU and Shannon index) was not significantly different from that of acute state patients (Fig. 1A). Bacterial composition in PCoA was not also significantly different between the two states, but there was much greater beta diversity in terms of weighted UniFrac distance in the acute state compared to the recovery state (Fig. 1B, C). Dysbiosis during the acute state may cause a more diverse distribution of gut microbiota. When relative abundances of the bacterial composition between the two states were analyzed at the family level, Enterobacteriaceae, Bacteroidaceae, Tannerellaceae, and Enterococcaceae tended to decrease, whereas Prevotellaceae, Lachnospiraceae, Oscillospiraceae, and Lactobacillaceae tended to increase after treatment (Fig. 2).

**Fig. 3:**
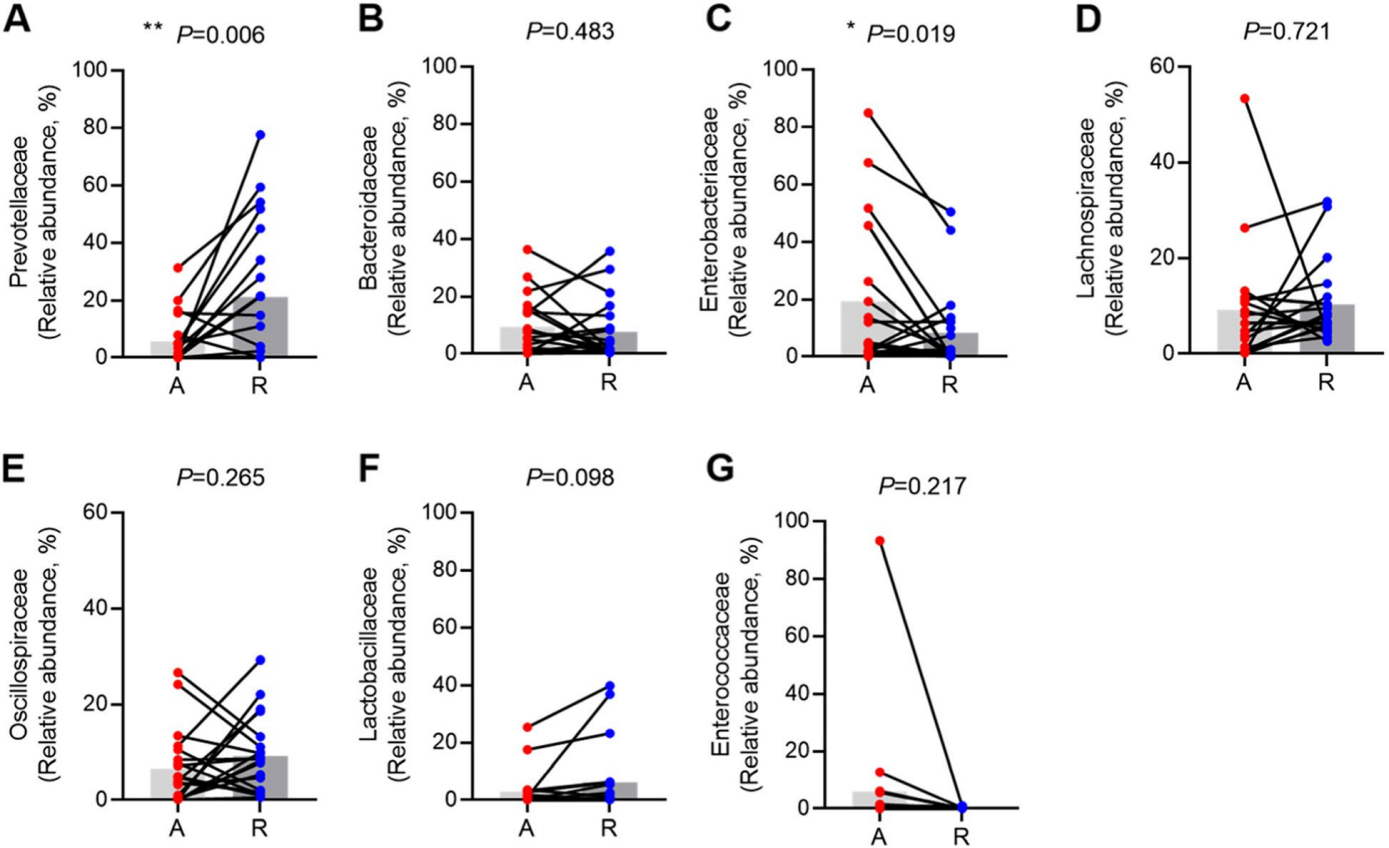
Comparisons of representative taxa at the family level between acute state and recovery state of gout. Prevotellaceae (**A**). Bacteroidaceae (**B**). Enterobacteriaceae (**C**). Lachnospiraceae (**D**). Oscillospiraceae (**E**). Lactobacillaceae (**F**). Enterococcaceae (**G**). The gut microbiota at the family level with more than 3% relative abundance were selected. * < 0.05; **P < 0.01 (paired *t*-test)

Paired *t*-test was performed to further investigate the differences in bacterial composition between both states. Compared to the acute state, the relative abundance of Bacteroidetes was increased in the recovery state, while that of Proteobacteria was decreased at the phylum level. The other dominant phyla such as Firmicutes and Actinobacteria were not significantly different between both states (Supplementary Fig. S1). Interestingly, Prevotellaceae (*p* = 0.006) and the genus *Prevotella* (*p* = 0.009) of Bacteroidetes were both significantly enriched in the recovery state, whereas Bacteroidaceae and the genus *Bacteroides* were not significantly different between both states. Among Proteobacteria, Enterobacteriaceae and its derivative genus *Shigella* were significantly decreased in the recovery state (*p* = 0.019 and *p* = 0.023, respectively) (Fig. 3, Supplementary Fig. S2). Although the derivatives of Firmicutes (i.e., Lachnospiraceae, Oscillospiraceae) were not significantly different between both states, the genera *Faecalibacterium* and *Roseburia*, which belong to Clostridiales, were significantly increased in the recovery state (Supplementary Fig. S2).

### Changes in serum SCFA levels after treatment in patients with gout

**Fig. 4:**
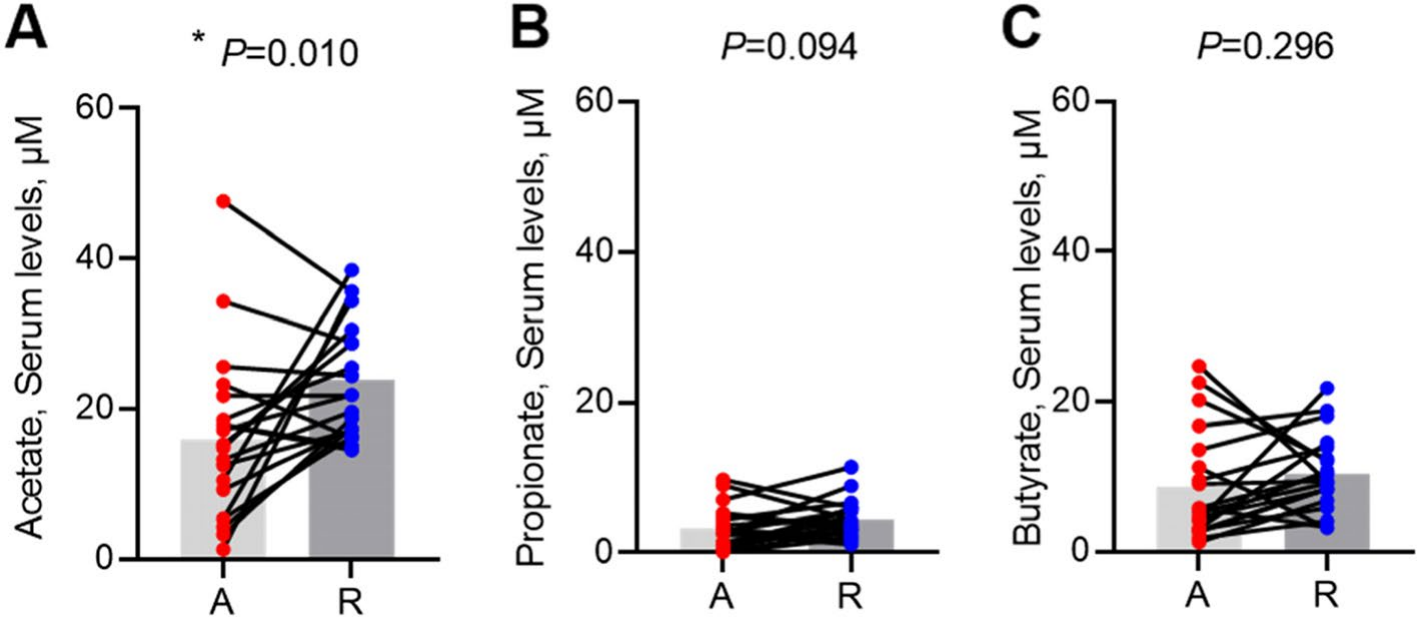
Changes in the levels of short-chain fatty acids between acute state and recovery state of gout. Acetate (**A**). Propionate (**B**). Butyrate (**C**). *P < 0.05 (paired *t*-test)

Many commensal gut microbiotas reportedly have anti-inflammatory effects through the production of SCFAs [4]. To explore the relationship between SCFA levels and inflammatory states of gout, the serum levels of SCFAs were compared between the acute and recovery states. As shown in Fig 4, acetate levels in the recovery state were dramatically increased compared to the acute state (*p* < 0.010). Propionate and butyrate levels tended to increase, but these were not significant.

## Discussion

The present study demonstrated two main results using serum SCFAs and fecal microbiota according to different inflammatory states (acute vs. recovery state) in the same patients with gouty arthritis. First, gut microbiotas were significantly altered, with an increased Prevotellaceae and decreased Enterobacteriaceae during the recovery state of gouty arthritis. Second, the production of SCFAs, especially acetate, was significantly decreased in the acute state but increased in the recovery state. Our results suggest that the recovery of inflammation in gouty arthritis may involve changes in the composition of gut microbiota and enhanced production of SCFAs.

Enterobacteriaceae were increased by more than 20% in the acute state of gout, indicating an increase during the inflammatory state because Enterobacteriaceae in feces of normal adults is usually around 5% [14]. Enterobacteriaceae induce interleukin (IL)-8 and IL-1β secretion and cause colitis with increasing intestinal inflammation [15]. Furthermore, the increased Enterobacteriaceae, which contains opportunistic pathogens such as *Salmonella*, *Shigella*, *Klebsiella*, and *E.coli*, could result in reduced or perturbed SCFA production which may initiate the host inflammatory response [16, 17].

An analysis of gut microbiota composition in the general population revealed three predominant variants (i.e., enterotypes), specifically *Bacteroides*, *Prevotella*, and *Ruminococcus*. Because *Ruminococcus* occupies the gut microbiota in low levels, the enterotype clustering was primarily composed of the genera *Bacteroides* and *Prevotella* [18, 19]. The *Bacteroides* enterotype was associated with animal protein and saturated fatty diets. In the *Prevotella* enterotype, healthy subjects exhibited improved glucose metabolism after consumption of kernel-based bread, and this was associated with carbohydrates and high-fiber diets [19, 20]. In our study, the relative abundances of Prevotellaceae and the other family of Bacteroidetes showed an inverse correlation. Interestingly, the ratio of the genera *Prevotella* and *Bacteroides* was less than 0.5 in the acute state but significantly increased to more than 0.5 in the recovery state (Supplementary Fig. S2F). This indicates that stable switching between the two enterotypes occurred after treatment of acute gouty arthritis in the same patients.

Previous studies also demonstrated that the genus *Bacteroides* was enriched in patients with gouty arthritis and that genera *Escherichia* and *Shigella* of the Enterobacteriaceae weres more abundant in those with tophi compared to the general population [21, 22]. The genus *Bacteroides* was associated with monocyte-derived cytokines (i.e., IL1-β and IL-6) and maintained epithelial barrier integrity by regulating intraepithelial lymphocytes (from which IL-6 is derived), suggesting that it could mediate a homeostatic role for the host immune system in the intestine [23, 24]. Therefore, the increase in Prevotellaceae alongside the relatively decreased Bacteroidaceae may be related to the recovery of acute gout arthritis.

The recent epidemiology of gout showed substantially increased prevalence and incidence worldwide, in close relationship with diet and behavioral changes. Especially, high fructose intake in sweetened beverages and insulin resistance, such as obesity, resulted in hyperuricemia and triggered gout flare in some people [1]. Feeding a high-sugar diet affects the gut microbial composition and depletes the concentration of intestinal acetate in mice, which is an attribute to increased susceptibility to chemically induced inflammatory colitis. In contrast, the supplementation of acetate attenuated high sugar-induced colitis [25]. SCFAs including oral acetate administration to mice fed a high-fat diet reduced obesity and improved insulin resistance by influencing energy expenditure without physical activity [26].

MSU crystals alone do not promote the inflammatory response of gouty arthritis; these require additional trigger factors which provide co-signals for the activation of macrophages. Particularly, free fatty acids and SCFAs from dietary intake could engage Toll-like receptor 2 and G-protein coupled receptor (GPR) 43 on macrophages, respectively, leading to the regulation of inflammation [2, 27]. Similar to our results, dietary fiber, which promotes the expansion of *Prevotella*, has been shown to increase the production of SCFAs. These SCFAs bind to the metabolite-sensing receptor GPR43 on the macrophage, which protected against diabetic nephropathy in mice [28]. Furthermore, supplement with acetate (a type of SCFA) has been found to induce faster resolution of inflammation in an experimental model of gouty arthritis, although it did not affect the onset of gout [5].

Because short-term diet changes are known to have little effects on enterotype clustering [15], the phenomenon in our study of switching to an enterotype which enhances SCFA production could be attributed to treatment with anti-inflammatory and urate-lowering agents. Further studies are needed to investigate whether long-term diets and manipulation of specific bacteria would affect the gut microbiota composition and the production of SCFAs, possibly preventing flares of gouty arthritis.

This study had several limitations. First, our study was conducted with relatively small participants of a single medical center, although some microbiotas and metabolites changed statistical significance. Second, healthy controls and comparative samples before the gout attack were not obtained. Therefore, it was impossible to determine whether the microbiome in the recovery state was completely recovered compared with before the gout attack. Third, several factors, including diet, lifestyle, and concomitant medications that could affect the microbiome, were not controlled. A well-designed multicenter prospective study is needed in the future. Finally, mechanical studies should be added on the causal relationship to determine whether changes in the gut microbiota contribute to reducing inflammation.

## Conclusion

In conclusion, the recovery state of gout arthritis had increased SCFA production genera (*Prevotella*, *Faecalibacterium*, and *Roseburia*) and fewer inflammatory-related genera (*Shigella*) compared with the acute state which resulted in the promotion of SCFA formation, especially acetate. Among the SCFA production strains, the rise of *Prevotella* was remarkable and was related to the change in the *Prevotella*/*Bacteroids* ratio. Further exploration of an axis involving the gut and joints and its mechanism may provide novel strategies for treating gouty arthritis by manipulating the gut microbiota and dietary intake.

## Supplementary Information

**Additional file 1**: **Figure S1**. Comparison of microbial composition between the acute state and recovery state at the phylum level. *: *P* < 0.05; **: *P* < 0.01 (paired t-test). **Figure S2**. Changes in bacterial taxa at the genus level between the acute state and recovery state. Prevotella (A), Bacteroides (B), Shigella (C), Faecalibacterium (D), Roseburia (E), Changes in the ratio of Prevotella and Bacteroides (F). A: Acute state; R: Recovery state; *: *P* < 0.05; **: *P* < 0.01 (paired t- test).

## Data Availability

The data sets generated and/or analyzed in the current study are publicly available.

## Abbreviations

MSU: Monosodium urate
SCFAs: Short-chain fatty acids
PCoA: Principal coordinate analysis
LEfSe: Linear discriminant effect size
LDA: Linear discriminant analysis
IL: Interleukin
GPR: G-protein coupled receptor

## References

[1] Pascart T, Lioté F (2019). Gout: state of the art after a decade of developments. Rheumatology (Oxford).

[2] Joosten LA, Netea MG, Mylona E, Koenders MI, Malireddi RK, Oosting M (2010). Engagement of fatty acids with Toll-like receptor 2 drives interleukin-1β production via the ASC/caspase 1 pathway in monosodium urate monohydrate crystal-induced gouty arthritis. Arthritis Rheum.

[3] Cleophas MC, Crişan TO, Joosten LA (2017). Factors modulating the inflammatory response in acute gouty arthritis. Curr Opin Rheumatol.

[4] Louis P, Hold GL, Flint HJ (2014). The gut microbiota, bacterial metabolites and colorectal cancer. Nat Rev Microbiol.

[5] Vieira AT, Galvão I, Macia LM, Sernaglia ÉM, Vinolo MA, Garcia CC (2017). Dietary fiber and the short-chain fatty acid acetate promote resolution of neutrophilic inflammation in a model of gout in mice. J Leukoc Biol.

[6] Wen X, Lou Y, Song S, He Z, Chen J, Xie Z, et al. Qu-Zhuo-Tong-Bi Decoction alleviates gouty arthritis by regulating butyrate-producing bacteria in mice. Front Pharmacol. 2021;11:610556. 10.3389/fphar.2020.610556 PMC7884811 33603667

[7] Neogi T, Jansen TL, Dalbeth N, Fransen J, Schumacher HR, Berendsen D (2015). 2015 Gout classification criteria: an American College of Rheumatology/European League Against Rheumatism collaborative initiative. Ann Rheum Dis.

[8] Gaffo AL, Schumacher HR, Saag KG, Taylor WJ, Dinnella J, Outman R (2012). Developing a provisional definition of flare in patients with established gout. Arthritis Rheum.

[9] Chen S, Zhou Y, Chen Y, Gu J (2018). fastp: an ultra-fast all-in-one FASTQ preprocessor. Bioinformatics..

[10] Magoč T, Salzberg SL (2011). FLASH: fast length adjustment of short reads to improve genome assemblies. Bioinformatics..

[11] Li W, Fu L, Niu B, Wu S, Wooley J (2012). Ultrafast clustering algorithms for metagenomic sequence analysis. Brief Bioinform.

[12] Zhang Z, Schwartz S, Wagner L, Miller W (2000). A greedy algorithm for aligning DNA sequences. J Comput Biol.

[13] Caporaso JG, Kuczynski J, Stombaugh J, Bittinger K, Bushman FD, Costello EK (2010). QIIME allows analysis of high-throughput community sequencing data. Nat Methods.

[14] Shin NR, Whon TW, Bae JW (2015). Proteobacteria: microbial signature of dysbiosis in gut microbiota. Trends Biotechnol.

[15] Seo SU, Kamada N, Muñoz-Planillo R, Kim YG, Kim D, Koizumi Y (2015). Distinct commensals induce interleukin-1β via NLRP3 inflammasome in inflammatory monocytes to promote intestinal inflammation in response to injury. Immunity..

[16] Litvak Y, Byndloss MX, Tsolis RM, Bäumler AJ (2017). Dysbiotic Proteobacteria expansion: a microbial signature of epithelial dysfunction. Curr Opin Microbiol.

[17] Ohira H, Tsutsui W, Fujioka Y (2017). Are short chain fatty acids in gut microbiota defensive players for inflammation and atherosclerosis?. J Atheroscler Thromb.

[18] Arumugam M, Raes J, Pelletier E, Le Paslier D, Yamada T, Mende DR (2011). Enterotypes of the human gut microbiome. Nature..

[19] Wu GD, Chen J, Hoffmann C, Bittinger K, Chen YY, Keilbaugh SA (2011). Linking long-term dietary patterns with gut microbial enterotypes. Science..

[20] Kovatcheva-Datchary P, Nilsson A, Akrami R, Lee YS, De Vadder F, Arora T (2015). Dietary fiber-induced improvement in glucose metabolism is associated with increased abundance of Prevotella. Cell Metab.

[21] Guo Z, Zhang J, Wang Z, Ang KY, Huang S, Hou Q, et al. Intestinal microbiota distinguish gout patients from healthy humans. Sci Rep. 2016;6:20602. 10.1038/srep20602 PMC4757479 26852926

[22] Méndez-Salazar EO, Vázquez-Mellado J, Casimiro-Soriguer CS, Dopazo J, Çubuk C, Zamudio-Cuevas Y (2021). Taxonomic variations in the gut microbiome of gout patients with and without tophi might have a functional impact on urate metabolism. Mol Med.

[23] Schirmer M, Smeekens SP, Vlamakis H, Jaeger M, Oosting M, Franzosa EA (2016). Linking the human gut microbiome to inflammatory cytokine production capacity. Cell..

[24] Kuhn KA, Schulz HM, Regner EH, Severs EL, Hendrickson JD, Mehta G (2018). Bacteroidales recruit IL-6-producing intraepithelial lymphocytes in the colon to promote barrier integrity. Mucosal Immunol.

[25] Laffin M, Fedorak R, Zalasky A, Park H, Gill A, Agrawal A (2019). A high-sugar diet rapidly enhances susceptibility to colitis via depletion of luminal short-chain fatty acids in mice. Sci Rep.

[26] Canfora EE, Jocken JW, Blaak EE (2015). Short-chain fatty acids in control of body weight and insulin sensitivity. Nat Rev Endocrinol.

[27] Vieira AT, Macia L, Galvão I, Martins FS, Canesso MC, Amaral FA (2015). A role for gut microbiota and the metabolite-sensing receptor GPR43 in a murine model of gout. Arthritis Rheumatol.

[28] Li YJ, Chen X, Kwan TK, Loh YW, Singer J, Liu Y (2020). Dietary fiber protects against diabetic nephropathy through short-chain fatty acid-mediated activation of G protein-coupled receptors GPR43 and GPR109A. J Am Soc Nephrol.

