# Supplementary material for: Treatment of gouty arthritis is associated with restoring the gut microbiota and promoting the production of short-chain fatty acids
Source: Arthritis Res Ther. 2022 Feb 19;24:51. doi: 10.1186/s13075-022-02742-9 (PMC8857835; doi:10.1186/s13075-022-02742-9)

**Supplementary fig. 1 Comparison of microbial composition between the acute state and recovery state at the phylum level.** ^*^: *P* < 0.05; ^**^: *P* < 0.01 (paired t-test).


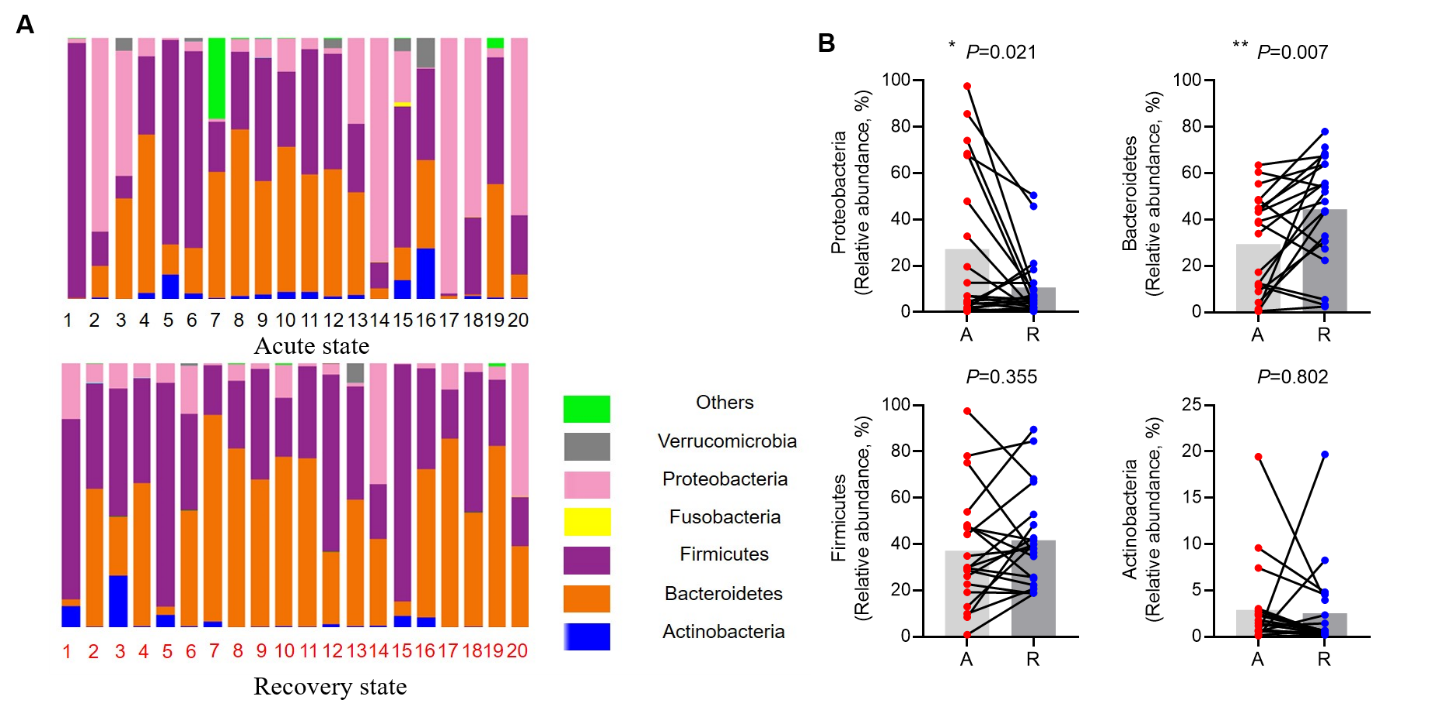


**Supplementary fig. 2 Changes in bacterial taxa at the genus level between the acute state and recovery state**. *Prevotella* (A), *Bacteroides* (B), *Shigella* (C), *Faecalibacterium* (D), *Roseburia* (E), Changes in the ratio of *Prevotella* and *Bacteroides* (F). A: Acute state; R: Recovery state; ^*^: *P* < 0.05; ^**^: *P* < 0.01 (paired t- test).


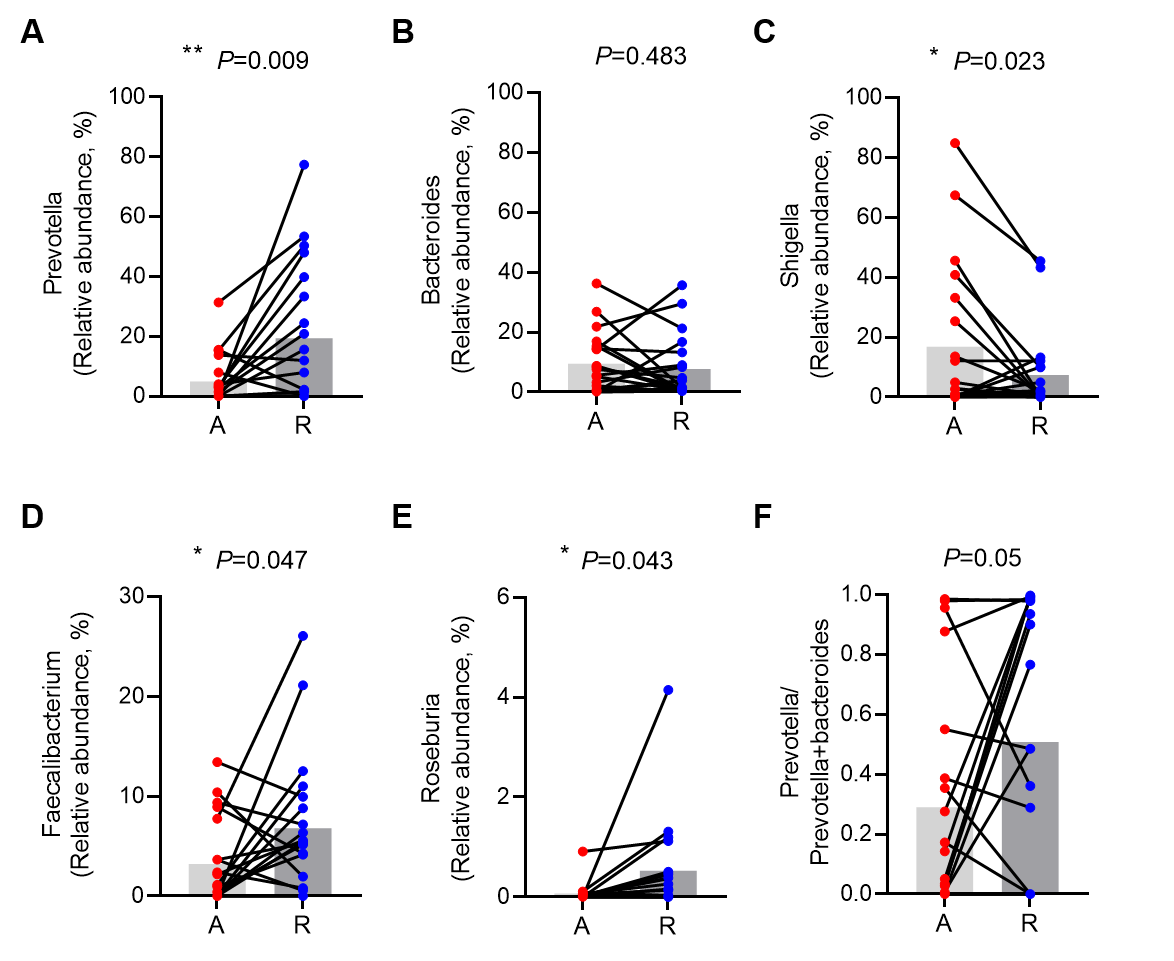

Supplement: Supplementary file 1 — Additional file 1: Figure S1. Comparison of microbial composition between the acute state and recovery state at the phylum level. *: P < 0.05; **: P < 0.01 (paired t-test). Figure S2. Changes in bacterial taxa at the genus level between the acute state and recovery state. Prevotella (A), Bacteroides (B), Shigella (C), Faecalibacterium (D), Roseburia (E), Changes in the ratio of Prevotella and Bacteroides (F). A: Acute state; R: Recovery state; *: P < 0.05; **: P < 0.01 (paired t- test). [file 13075_2022_2742_MOESM1_ESM.docx]
